# Is *hybrid*-PBL advancing teaching in biomedicine? A systematic review

**DOI:** 10.1186/s12909-019-1673-0

**Published:** 2019-06-24

**Authors:** Rodrigo Jiménez-Saiz, Domenico Rosace

**Affiliations:** 1 Department of Biochemistry and Molecular Biology, Chemistry School, Complutense University, Ciudad Universitaria s/n, 28040 Madrid, Spain; 2 McMaster Immunology Research Centre (MIRC), Department of Pathology and Molecular Medicine, McMaster University, Hamilton, ON Canada; 3 Epitranscriptomic and Cancer Lab, Cancer Research Center (CIC), Miguel de Unamuno University, 37007 Salamanca, Spain

**Keywords:** Hybrid problem-based learning, Hybrid-PBL, Biomedicine, Systematic review, Higher education

## Abstract

**Background:**

The impact of instructional guidance on learning outcomes in higher biomedical education is subject of intense debate. There is the teacher-centered or traditional way of teaching (TT) and, on the other side, the notion that students learn best under minimal guidance such as problem-based learning (PBL). Although the benefits of PBL are well-known, there are aspects susceptible to improvement. Hence, a format merging TT and PBL (*hybrid-*PBL, h-PBL) may advance education in biomedical sciences.

**Methods:**

Studies that employed h-PBL in higher biomedical education compared to TT and/or pure PBL were systematically reviewed. Specifically, this review addressed the following question: does h-PBL in biomedical sciences result in superior marks and a better student’s perception of the teaching and learning process?

**Results:**

We found that the use of h-PBL in higher biomedical sciences was superior compared to TT and pure-PBL. This was evidenced by the higher performance of the students in h-PBL as well as the level of student’s satisfaction as compared to TT or pure PBL.

**Conclusions:**

These findings encourage more research on investigating the pedagogical benefits of h-PBL. In addition, these data support an eclectic system in which the pedagogical tools from TT and PBL are used cooperatively in the best interest of the education and satisfaction of the students.

## Background

Education is a fundamental component of the decision-making process of every individual (and by extension of a society), which is essentially based on the acquisition and critical use of knowledge. Therefore, the method employed to educate (i.e. teach) profoundly impacts the social, cultural and professional endeavours of every person. On this note, there has been a heated debate on the impact of instructional guidance on learning outcomes (e.g. knowledge retention, critical thinking, communication and practical skills, etc.) for more than 50 years, particularly as it pertains to higher education [1–3].

On one side, there is the teacher-centered or traditional way of teaching (TT), in which there is direct instructional guidance on the concepts and procedures required by a given discipline; that is to say that there is direct transmission of knowledge from the instructor to the students. In this method, the instructor completely controls the teaching agenda as he decides what concepts and skills need to be learnt as well as the sequence, the pace and the style of teaching it. While this method ensures, a priori*,* a homogeneous transmission of key knowledge to all the students, it does not adapt well to the background and learning abilities of them, which are usually heterogeneous. This modality of teaching often involves large-classes and lecture-based deliveries [4, 5].

On the other side, there is the notion that students learn best in a minimally guided or unguided environment including problem-based-learning (PBL), inquiry-based learning, project-based learning and discovery-based learning. These types of teaching are closer to an inductive reasoning, where one goes from an event to a conclusion that could, eventually, become a general statement. Of these educational methods, PBL has been increasingly adopted since its introduction in 1969 in the Medical Sciences program of McMaster University [6]. PBL is defined as a learner-centered approach that empowers small groups of students to conduct research, integrate theory and practice, and apply knowledge and skills to develop a viable solution to a defined problem [4]. The student needs to be active and take full responsibility for his own learning, thus fostering self-learning skills. This can be, at times, challenging if students lack discipline and/or feel overwhelmed directing and structuring their own learning [4, 7].

Since its introduction, PBL has been linked to several theories [8]. Constructivists suggest that new knowledge is built on prior knowledge and experience [9, 10] and that an active process of learning is needed for knowledge acquisition [11]. It could be argued that PBL is a constructivist pedagogy in which students learn and develop critical thinking skills by solving real-world problems in small groups [12]. Moreover, PBL is an active learning method that stimulate students to interact and experience. Experiential learning identifies experience, observation and reflection as the foundation and stimulus for learning [13, 14]. Hence, the four-stage learning cycle (experience, reflection, analysis and conclusions) is related to PBL as a method that is functional to the student’s growth and maturation [15]. In addition, PBL is related to social and collaborative theories. The first one posits learning as a social process that includes interactions and active engagement [16]; the basis of the second is collaboration, engagement and student’s group work for active learning [17]. As in PBL, collaborative learning considers the need to learn together through mutual interactions and shared understanding [18]. Finally, PBL shares similarities with the cognitive theory. Students are prone to learn when intellectually active and engaged with the learning process [19]. This is particularly relevant for problem analysis, an important phase of PBL [20] based on discussion and group interaction to define a solution [21]. PBL is therefore based on principles common to many learning theories that reflect and guide the way PBL is implemented.

There is increasing advocacy towards the use of PBL in higher education in various fields, including biomedical sciences, under the premise that PBL is a (if not “the”) superior way of teaching; indeed, to teach any other way may be even considered unethical [3]. However, the evidence to support an absolutist view is debatable [1, 2]. While it is plausible that PBL, or pedagogically comparable teaching methods (e.g. experiential, discovery, inquiry-based learning), are pivotal for higher teaching [3], a number of studies have identified aspects of PBL that are susceptible to improvement [2, 22, 23]. For example, PBL instructors often witness students that are stuck with a problem [23], which raises the need to tailor PBL to the knowledge of the students and complement it with guided sessions (e.g. lectures). Indeed, medical students (from 1st and 2nd year) in the UK [24], and a vast majority of dental students (from 2nd to 5th year) in PBL-based programs from USA or Sweden [7], wanted lectures, at least sometimes, which indicates a need for more guidance. This suggests that lecture-based, guided sessions may be a useful teaching tool to fulfill certain deficiencies of the PBL-curriculum. In other words, that a *hybrid-*PBL (h-PBL) format that incorporates elements of TT and PBL may advance teaching and education in biomedical sciences.

The concept of h-PBL, understood as a combination of PBL and TT, is not unforeseen [25]. In fact, it has been used in a number of biomedical programs that were transitioning from TT to PBL, in programs where TT is deeply rooted and the faculty members would not support a pure PBL system, and it has also been employed by instructors genuinely interested in investigating the learning outcomes of h-PBL [26–29]. While some studies have tested h-PBL in biomedical sciences, a comprehensive analysis and review of the data generated has not been performed, which makes it difficult to define accurately its pedagogical value. Here, we have conducted a systematic review of experimental studies that employed h-PBL in higher biomedical education compared to TT and/or pure PBL. Specifically, this review addresses the following question: does h-PBL in biomedical sciences result in superior marks and a better student’s perception of the teaching and learning process?

## Methods

**Fig. 1:**
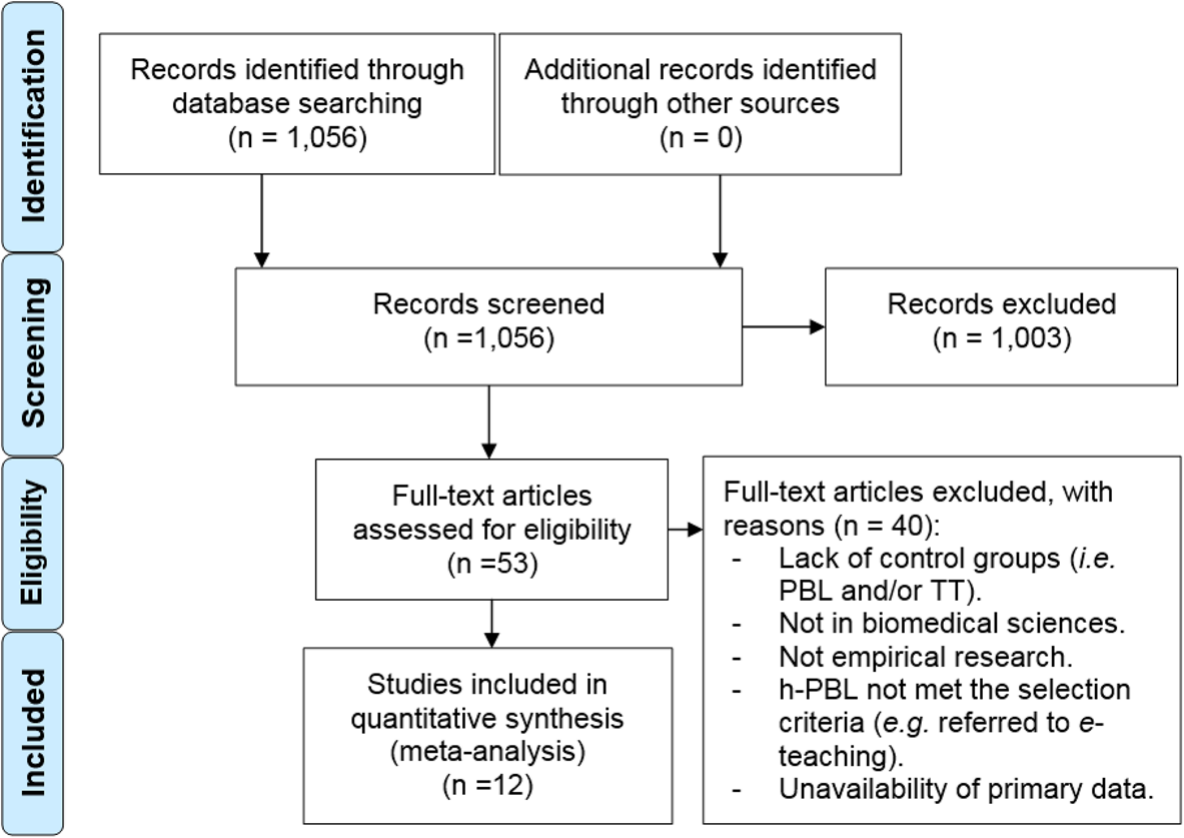
PRISMA flow diagram demonstrating identification and screening stages and included articles

This study was conducted following the Preferred Reporting Items for Systematic Reviews and Meta-Analyses (PRISMA) guidelines to systematically and explicitly screen studies in a rigorous and unbiased manner [30]. The PRISMA flow diagram (Fig. 1) conveys the different phases of this systematic literature review from the number of records identified through to those included and excluded (with reasons). Data were collected from original research in higher education, in biomedical sciences, involving a h-PBL group and a TT and/or pure PBL group. Articles published in peer-reviewed academic journals between 1997 and January 2018 were examined. With the support of the staff from the Paul R. McPherson Institute for Leadership, Innovation, Excellence in Teaching at McMaster University (Hamilton, ON) databases were selected to find original research on h-PBL. A keyword search was conducted in 3 databases including ERIC, Web of Science and PubMed. The search terms were discussed and agreed upon by all authors to ensure relevant articles were located. For the purposes of this systematic review, the important search terms were: ‘hybrid-problem-based learning’, ‘hybrid-PBL’ and related terms. These search terms were applied for each of the 3 databases separately and records found were pooled using EndNote7.

The initial search of three databases identified 1056 records (Fig. 1). These records were screened by reading the title and abstract. At this screening stage, records were excluded if they were (a) duplicates, (b) not in English, (c) not in higher education, (d) not in biomedical sciences, (e) not original research. Following this initial screen, 53 records remained, which were then assessed for eligibility (Fig. 1). More detailed inclusion criteria were then applied to these articles. Articles were excluded for the aforementioned reasons, and also if they a) did not include control groups or b) the h-PBL system used was referring to *e*-teaching. After assessing the 53 full text articles, 12 articles fitting the eligibility criteria remained and these were analyzed in the review (Table 1).

**Table 1:** Studies selected following the PRISMA guideline

Study	Country	Discipline	Groups	Total n number	Factual knowledge	Problem-solving skills	Student’s perception
[26]	Spain	Biology	h-PBL, TT	85	Yes	No	No
[28]	USA	Medicine	h-PBL, TT	71	Yes	Yes	Yes
[29]	Canada	Pharmacy	h-PBL, TT	64	Yes	Yes	Yes
[31]	Spain	Biology	h-PBL, TT	60	Yes	No	Yes
[32]	China	Medicine	h-PBL, TT	273	Yes	No	Yes
[33]	China	Medicine	h-PBL, TT, PBL	127	Yes	Yes	Yes
[34]	USA	Physiology	h-PBL, TT	187	Yes	No	Yes
[35]	USA	Chemistry	h-PBL, TT	300	No	Yes	Yes
[36]	India	Medicine	h-PBL, TT	118	No	No	Yes
[37]	China	Medicine	h-PBL, TT	205	Yes	Yes	Yes
[38]	USA	Biology	h-PBL, TT	–	No	No	Yes
[39]	Turkey	Medicine	h-PBL, TT, PBL	547	No	No	Yes

## Results

### Characterization of selected articles

The 12 original research studies on h-PBL in higher biomedical sciences that were selected for the analysis are summarized in Table 1.

**Fig. 2:**
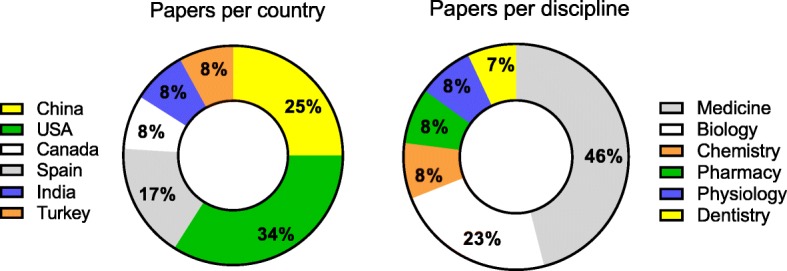
Country of origin and disciplines of the articles selected

The majority of articles were originated in the USA and China (34 and 25% respectively), followed by Spain (17%), and Canada, India and Turkey (8% each) (Fig. 2). At the continent level, 42% of the selected articles were from Asia and North America each, and 16% from Europe.

Medicine was the discipline that prevailed in the selection (46%), followed by biology (23%), physiology, chemistry and pharmacy (8% each), and dentistry (7%) (Fig. 2). These data indicate that the findings of this systematic review are especially representative of the field of medicine in USA and China.

All the selected studies included an appraisal of student performance that evaluated theoretical knowledge and/or problem-solving skills as well as an evaluation of student’s perception of the course. We considered that an objective evaluation of the knowledge and practical skills developed by the students, in combination with their personal perception and satisfaction of the course, would provide representative readouts of the overall pedagogical benefits obtained with each teaching system.

Most of the studies compared 2 groups (10 articles, 83%), and 2 articles (17%) had the ideal design comparing the 3 groups of interest (h-PBL, pure PBL and TT). Furthermore, the selected articles for the present study compared h-PBL vs TT (9 articles, 75%) more often than h-PBL vs PBL (3 articles, 25%). This implies that the findings of this systematic review are more substantiated when comparisons between h-PBL and TT are made. Due to the limited number of studies comparing h-PBL vs PBL, or the 3 groups, these studies will be analyzed in more detail.

### Students performance

Grades are usually taken as an indicator of student performance. To determine whether students in a h-PBL program performed better compared to TT and/or PBL, academic records were compared. Grading was assigned based on factual knowledge and/or problem-solving skills. To assess theoretical knowledge, students belonging to different teaching methods performed a test that took place at the end of the semester, or the year, centered on basic science knowledge acquired during this time frame. The exams consisted of multiple-choice questions, short-essay questions, or a combination of both. To assess problem-solving skills, students were to solve a problem-based or a case-study exam; science comprehension, diagnosis, treatment, communication and hypothesis generation, among others, were evaluated.

**Fig. 3:**
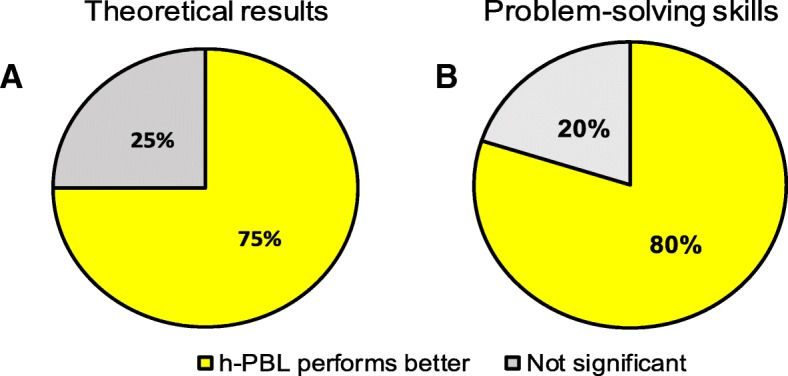
Students performance pertaining to theoretical results (A) and problem-solving skills (B) in h-PBL compared to TT

Eight out of the 12 articles selected (66%) analyzed student’s theoretical knowledge and 5 (42%) of them assessed problem-solving skills (Table 1). Students in the h-PBL group obtained better theoretical results compared to TT (*p* < 0.05) (Fig. 3a). Six studies (75%) showed significantly better performance of students in the h-PBL compared to TT (*p* < 0.05) and 2 studies (25%) did not show significant differences. In these 2 studies [29, 31], the students belonging to the h-PBL program had similar scores to the other experimental groups and did not show learning shortcomings. Interestingly, the study by Carrió et al. [31], which compared 2nd year students educated with TT or hybrid-PBL, did not show significant differences in factual knowledge acquisition between both groups at that time. However, in a follow up study with the same cohort of students, upon completion of the degree (5 years), Carrió et al. [26] reported that h-PBL students obtained higher marks than TT students, which suggests that h-PBL improved long-term retention of knowledge. In addition, students in the h-PBL group demonstrated better problem- solving skill performance compared to TT in 80% (4 out of 5) of the studies (Fig. 3b).

We found 2 studies comparing h-PBL and pure-PBL learning methods although only one compared student’s performance [33]. This recent study was conducted by the Department of Neurology at Sun Yat-Sen Memorial Hospital of China, with 127 students from a five-year undergraduate program that voluntarily participated*.* The aim of the authors was to introduce h-PBL in neurology and compare student’s performance to pure PBL and TT. The students were randomly assigned to each group and their performance was evaluated with a theoretical and a practical test at the end of the course. The test addressed the students’ understanding of the fundamental concepts taught as well as the diagnosis and treatment for the diseases covered in the course. The practical test mainly evaluated the students’ proficiency analyzing a medical case, formulating a hypothesis and a strategy for the physical examination. They found that the PBL group performed better in the practical test, while the theoretical test scores and the total scores of the h-PBL students were significantly higher than those of the other groups. Interestingly, the differences in scores were greater when comparing the results of the h-PBL vs the TT group.

### Students perception

The perception of students was also analyzed as indicator of their satisfaction with the pedagogical method employed in the course. A questionnaire to investigate students’ preference for either h-PBL, PBL or TT was performed after the course. The questionnaires were specific for each study but they evaluated student’s satisfaction on common aspects such as learning, comprehension, interest, motivation and personal/technical skills acquired with the teaching method (Table 2). An example of the questions asked is given below:

- if you had the possibility to choose before the course, would you have opted for the PBL course/h-PBL course/TT course?
- after the experience from the course, would you now opt for the h-PBL course if you had to choose again?
- which kind of teaching method is the more appropriate and supporting to achieve the key learning objectives planned at the beginning of the course?

**Table 2:** Identification of students’ perception

Study	Specifics
[26, 31]	Students rated, from 0 to 10, the acquisition of a list of generic skills developed during the course (hypothesis development, argumentation, synthesising, data analysis, communication skills, time management, cooperative working, etc.)
[29]	Questionnaire taken at the time of graduation and 1 year after with 24 questions on demographics, College of Pharmacy and practice experience, educational preparation and opinions about the overall educational experience and learning methods.
[32]	Students answered “agree”, “neutral” or “disagree” to 7 questions regarding the effectiveness of the teaching method and student’s attitude and satisfaction. Results were statistically analyzed.
[33]	Student’s satisfaction was evaluated for various aspects (e.g. motivation to prepare before class, ability of thinking, activating class atmosphere, teamwork spirit, problem-solving skills, understanding of difficult topics, etc.) in a 4-point-scale: excellent, good, fair and poor.
[34]	An online survey evaluated student’s perception regarding the teaching strategies.
[35]	At the end of each semester, students answered 3 questions on a scale from 1 to 7 on their perception of learning and satisfaction: I am satisfied with what I learned in this course; on a scale of 1 to 7 this course was…; I learned a good deal of factual material in this course.
[36]	A Likert 5-point scale was used to assess students’ perception on 4 aspects: learning/understanding, interest/motivation, training one’s personal abilities and satisfaction, and confidence acquired. Results were statistically analyzed.
[37]	Two questions were asked to investigate student’s preference: if you had the possibility to choose before the course, would you have opted for the h-PBL course or lectured-based? After the course, would you now opt for the h-PBL or lecture-based course if you had to choose again? A second questionnaire assessed student’s satisfaction with the h-PBL course (content, framework, subjective effects, etc.). It included 20 questions that were answered using a Likert 5-point scale. The students also had the option to comment on the contents of the tutorial as well as on possibilities for improvement in full text.
[38]	Student’s satisfaction and perception of the course was assessed based on the feedback given to the instructors as well as the student’s retention rate during 2 semesters.
[39]	Two questionnaires (at the beginning of the 1st academic year and at the end of the 2nd) assessed the student’s learning preferences, and the students were classified according to 4 kinds of learners: assimilators, convergers, divergers and accommodators.

In addition, students had the opportunity to comment on the contents of the tutorial, as well as express their opinions and suggestions to improve the course [26, 33, 35, 37].

In all the articles that analyzed student’s perception the students provided positive feedback and the questionnaires showed higher average scores for h-PBL than TT or pure PBL. In particular, the students were satisfied with the h-PBL format because they considered that this method helped them learn relatively complex and nonintuitive parts of the program more easily than with pure PBL. They also noted the cooperative work and informational skills [26] and the ability of thinking independently and critically [35] as reinforcements of the acquired skills in h-PBL. In the study by Yang et al., [33] the questionnaire conducted on the h-PBL and TT groups, showed that some students had negative feedback on pure PBL, which was mainly due to students’ difficulties to gain a comprehensive understanding of the subjects. On the other hand, the h-PBL method was widely accepted by the students, achieving 100% of satisfaction and preferences. Lastly, Gupinar et al. [39] conducted a prospective study to evaluate the perception of medical students about their learning style. Although learning styles are discredited [40, 41], the majority of 1st and 2nd year medical students (~ 88%) fitted an assimilator (e.g. instructor-based) or a converger profile (e.g. PBL), which presumably would benefit from an h-PBL method.

## Discussion

The overarching goal of this systematic review was to advance higher education in biomedical sciences by questioning current views that promote the exclusive use of pure TT or PBL [1–3]. We hypothesized that a h-PBL format that incorporates elements of TT and PBL may benefit the students pedagogically more than pure TT or PBL alone. We conducted a systematic literature review to compare the performance and/or perceptions of students in a h-PBL vs TT and/or PBL format in higher biomedical sciences. Specifically, this review addressed the following question: does h-PBL in higher biomedical sciences result in superior marks and student’s perception of the learning process?

Overall, this systematic review indicates that the use of h-PBL in higher biomedical sciences was superior compared to TT and pure-PBL. This is evidenced by the higher performance of the students in h-PBL as well as the level of student’s satisfaction. The better performance of h-PBL students, compared to pure PBL students, may be due to the insufficient guidance often felt by PBL students, which causes anxiety, struggling with certain problems, absence of a higher understanding of the field, etc. [7, 22, 23, 29]. Expectedly, the differences observed between h-PBL and TT students were more pronounced than when comparing h-PBL and PBL. This is likely due to the pedagogical benefits of problem-solving activities, which empower rationalization and long-term retention of knowledge [1].

It is reasonable to think that non-biomedical disciplines that require theoretical and practical knowledge would also benefit from a h-PBL format. In fact, some studies have reported benefits in using h-PBL in the fields of engineering [42, 43], foreign-language study [44] and business education [45] among others. From a geographical perspective, the majority of the studies analyzed originated from North America, Asia and, to a lesser extent, Europe. This geographical diversity, while limited, encompasses academic institutions ranging from highly experienced in PBL, in which PBL is well-established, to those that are prone to TT and completely naïve to PBL. Therefore, this systematic review provides a realistic, global view of the current acceptance of h-PBL by students and instructors as well as its pedagogical value.

While the results of this systematic review support the use of h-PBL in higher biomedical sciences over TT and PBL, there are limitations that need to be taken into consideration. For example, the low number of studies, particularly those directly comparing PBL and h-PBL, prevent us from giving strong recommendations. This systematic review is rather preliminary, but the findings clearly encourage more research on investigating the pedagogical benefits of h-PBL, and further studies in which PBL and h-PBL are directly compared and learning outcomes comprehensively analyzed.

Another limitation is that the questionnaire assessing student’s perception was not the same across the selected studies. Hence there may be biases on the importance that each study gave to the main areas evaluating student’s perception (i.e. learning and understanding; interest and motivation; training one’s personal abilities and satisfaction; confidence acquired with the teaching method). More importantly, the training and expertise in PBL of the instructors participating in these studies need to be carefully evaluated when designing the studies [46]. We found limited information about instructors’ experience in the selected studies. Whelan et al. [29] reported that all tutors attended a 2-day standardized tutor training program and they were observed by a peer during the full teaching period. Furthermore, they were part of a team composed of faculty, staff, students and stakeholders responsible of facilitating students’ transition from TT to h-PBL and stimulated them to use self-directed learning. However, instructors willing to investigate novel pedagogical methods often face the stagnation of other faculty members, their reluctance to prepare themselves to educate in a different format, and a lack of pedagogical and human resources in their departments. As such, some of the studies discussed here could not incorporate more than 20% of PBL teaching within the h-PBL program [26, 31] because of the aforementioned reasons.

There are additional aspects that are worth considering as they may have impacted the outcome of studies assessing the pedagogical value of h-PBL (and PBL). For example, how advanced the students are in their degree may influence their learning outcomes in h-PBL and PBL; the stronger background and maturity of senior students is a plus when student-centered teaching methods are applied. In addition, how familiar the students are with the methodology, and the duration of the study, may impact their predisposition towards it. This leads us to a different question; what should be the flavor of an h-PBL course? In other words, how many teaching hours should be delivered as PBL? It depends on a number of variables including the background and number of the students, their level of conceptualization, and their progress, to mention a few. Therefore, the ability of the facilitator to perceive learning hurdles as they arise, and switch from one format (less guidance, PBL) to another (more guidance, lecture-based) ad hoc is critical to maximize the potential benefits of h-PBL. This may be accomplished via regular assessment of students’ progress in a manner that comprehensively informs of the learning outcomes. Other aspects that need to be recognized when implementing h-PBL include the adequate training of instructors for this teaching method, the importance that students have a clear understanding of the h-PBL system (e.g., goals of h-PBL, functioning structure, expectations, etc.), and the design of exams intended to evaluate the acquisition of concepts as well as their application and practical skills.

## Conclusion

In conclusion, our findings refute an absolutist view on teaching in higher biomedical sciences and rather posit an eclectic system in which the pedagogical tools from TT and PBL are used cooperatively and in the best interest of the education and satisfaction of the students.

## Data Availability

Not applicable.

## Abbreviations

h-PBL: Hybrid problem-based learning
PBL: Problem-based learning
TT: Traditional teaching
